# Current concepts and challenges in pediatric diastolic dysfunction and heart failure with preserved ejection fraction

**DOI:** 10.1038/s41390-026-05038-9

**Published:** 2026-05-20

**Authors:** Luke D. Losordo, Ghadir Amin, Madhav Singla, Sheila Belk, Kenneth R. Butler, George W. Booz, Angela Monafo

**Affiliations:** 1 Department of Pharmacology and Toxicology, School of Medicine, The University of Mississippi Medical Center, Jackson, MS, USA; 2 Division of Cardiology and The University of Mississippi Medical Center, Jackson, MS, USA; 3 Department of Pediatrics, Division of Critical Care, The University of Mississippi Medical Center, Jackson, MS, USA; 4 Department of Medicine, University of Mississippi Medical Center, Jackson, MS, USA

## Abstract

Heart failure with preserved ejection fraction (HFpEF) has transitioned from an underrecognized and poorly characterized condition to a recognized, increasingly prevalent form of heart failure in the adult population, accounting for approximately half of all heart failure cases. Improved diagnostic capabilities and the growing burden of risk factors, including aging, hypertension, diabetes, and obesity, drive this rise. In contrast, pediatric HFpEF remains largely under-characterized, arising in a heterogeneous context that includes congenital heart disease and restrictive cardiomyopathies. The true incidence and prevalence of pediatric HFpEF remain difficult to establish due to challenges such as the absence of standardized diagnostic criteria, limited sensitivity of conventional diastolic function parameters, a lack of age-specific reference ranges for imaging and biomarker thresholds, and nonspecific symptoms that complicate early detection. While recent pharmacological advances in adult HFpEF, such as sodium-glucose cotransporter-2 inhibitors (SGLT2i) and glucagon-like peptide-1 (GLP-1) receptor agonists, have demonstrated incremental benefits, there is a notable absence of evidence-based treatments for pediatric HFpEF. This review provides an up-to-date overview of pediatric HFpEF, highlighting key diagnostic challenges and therapeutic gaps, discussing recent advancements in diagnostics and emerging therapeutic strategies with potential relevance for pediatric care.

## INTRODUCTION

The cardiac cycle is a coordinated sequence of contraction (systole) and relaxation (diastole) of the heart that efficiently pumps blood to the body. During diastole, the ventricles fill with blood. In early diastole, all chambers are relaxed, pressure drops, the AV valves open, and blood flows passively from the atria into the ventricles.^1^ In late diastole, the atria contract and push the remaining blood into the ventricles. Adequate blood delivery to the body is impaired if the ventricles, in particular the left ventricle, inadequately relax (due, for instance, to increased fibrosis or titin stiffness) or the size of the chamber is reduced because of concentric remodeling. Diastolic dysfunction is classified as the prolongation, incompletion, or slowing of ventricular relaxation and filling.^2^ Left ventricular diastolic dysfunction (LVDD) can be seen as the upward shift of the end portion of the left ventricular (LV) pressure-volume diagram, indicating that filling pressures are increased disproportionately to LV dilation. This can commonly cause symptoms of pulmonary congestion during exertion.

HFpEF is defined as heart failure (HF) with an ejection fraction (EF) of 50% or greater at diagnosis. While LVDD is considered a central diagnostic criterion for HFpEF, patients with HFpEF exhibit a heterogeneous array of systemic etiologic risk factors. As described by Borlaug in 2014, the pathophysiology of HFpEF involves limitations in ventricular diastolic, systolic, and chronotropic reserve, which interact with underlying factors such as the vasculature, endothelium, autonomic nervous system, and skeletal muscle.^3^

In adults, HFpEF is a heterogeneous syndrome characterized by preserved systolic function and often driven by metabolic, hemodynamic, and pro-inflammatory alterations. Accurate diagnosis requires consideration of conditions that can mimic HFpEF, including infiltrative, hypertrophic, or restrictive cardiomyopathies, valvular or pericardial disease, and other systemic or genetic disorders.^4^ In contrast, pediatric HFpEF is most commonly associated with congenital heart disease, though adult-like mimics may also contribute, underscoring the need for cautious interpretation of diastolic dysfunction. Within pediatric HFpEF, CHD was found in one study to make up 69.1% of pediatric HFpEF cases, followed by 7.4% cardiomyopathy cases.^5^

There has been substantial research and experimentation into adult HF. Heart failure with reduced ejection fraction (HFrEF) has seen significant scientific breakthroughs regarding effective treatment modalities.^6^ HFpEF clinical trials, on the other hand, have been largely unsuccessful, until recently, in identifying effective treatments for the disease.^7^ HF in children, specifically HFpEF, has different precipitating factors as compared to adults. Etiologies such as cardiomyopathies, congenital heart disease (CHD), chemotherapy, HIV, kidney failure, and obesity, among others, have been described as predictors of pediatric HFpEF. Sex differences in pediatric HFpEF patients are not well-documented; however, studies in related pediatric cardiac conditions suggest differences exist. While adult HFpEF predominantly affects females, studies in pediatric conditions such as non-syndromic hypertrophic cardiomyopathy show a male predominance (67% in one study), suggesting potentially different, age-specific drivers.^8^ Cardiac arrhythmias are relatively common in children with HF, with one study of 1228 pediatric heart failure patients identifying arrhythmias in 6.9% of the 814 HFpEF cases.^9^ Notably, the primary causes of mortality in HFpEF differ significantly between pediatric and adult patients. While adult mortality is largely driven by aging, hypertension, and metabolic comorbidities, pediatric HFpEF mortality is primarily driven by structural congenital heart disease and genetic cardiomyopathies.^10,11^

The etiologies, risk factors, clinical course, biomarkers, and therapies in children with HFpEF may differ not only from those in adults but also among children with the same diagnosis.^12^ Due to this heterogeneity, treatment and diagnostic options are limited in pediatric HFpEF. This review focuses on pediatric HFpEF, emphasizing recent developments in diagnostic modalities and treatment strategies, and highlighting the need for age-specific approaches in addressing this complex condition. The literature on HFpEF in children remains sparse. As a result, much of the current understanding is derived from adult HFpEF studies and pediatric HFrEF data. This review presents the currently available evidence, acknowledges the limitations of extrapolating across populations, and highlights critical knowledge gaps that warrant future pediatric-focused research.

## CLINICAL EVALUATION

### Diastolic dysfunction

Diastolic dysfunction or impaired relaxation during the filling phase of the LV is a hallmark of HFpEF, leading to a reduction in cardiac output. In general, in healthy children, diastolic dysfunction is not common but is frequently present in specific pediatric conditions like septic shock, CHD, and cardiomyopathies.^5,13,14^ A 2021 study found that when applying adult criteria, the prevalence was low (1.2–2.7%), but using pediatric-specific criteria, it ranged from 19–28% in youth with obesity, highlighting the importance of age- and population-specific standards for diagnosis.^15^

Diastolic dysfunction can be challenging to assess in children because of significant variability and inconsistency in echocardiographic criteria.^12^ Ventricular stiffness raises LV end-diastolic pressure and left atrial (LA) pressure, and the latter can increase LA size, which is often a characteristic feature of HFpEF.^16^ Conventional echocardiographic parameters may lack sensitivity in pediatric patients, but novel echocardiographic measures such as strain and strain rate analysis by speckle-tracking echocardiography and tissue Doppler imaging may help to define diastolic dysfunction in pediatric patients better.^12^

It is important to emphasize that while diastolic dysfunction is a hallmark of HFpEF, it is not specific to the syndrome. In adults, diastolic dysfunction can also be observed in conditions such as HFrEF, hypertension, diabetes, hypertrophic cardiomyopathy, and coronary artery disease. In children, HFpEF is most commonly associated with congenital heart disease and pediatric cardiomyopathies. Therefore, reliance on diastolic function alone is insufficient for diagnosis.

### Biomarkers and molecular mechanisms

Circulating biomarkers for HFpEF include NT-proBNP, a marker of cardiac stress, along with markers of fibrosis and inflammation, such as syndecan-1, C-reactive protein (CRP), interleukin 6, monocyte chemoattractant protein-1, galectin-3, and growth differentiation factor-15.^17,18^ Notably, in HFpEF, levels of NT-proBNP may be lower in individuals with obesity due to an impediment of myocardial stretch and changes in proBNP processing.^19^ Recently, there has been some interest in the profiling of circulating non-coding RNAs as indicators of fibrosis and HF (both HFrEF and HFpEF) severity.^20^

The molecular basis of diastolic dysfunction in pediatric patients remains poorly understood. In adults, HFpEF has been attributed to both fibrosis and increased stiffness of the sarcomeric protein titin due to a modified phosphorylation state.^21^ These events seem to be associated with coronary microvascular inflammation, at least in cases marked by comorbidities, such as obesity, hypertension, and diabetes mellitus.^22^ Alterations in mitochondrial function and associated Ca^2+^ handling may contribute as well to impaired diastolic function.^23^

### Diagnostic algorithms and limitations in pediatrics

Diagnostic algorithms such as the H_2_FPEF and HFA-PEFF scores combine echocardiography with biomarkers, clinical history, and other assessments to improve specificity. The H_2_FPEF score includes the following six variables: Body Mass Index (BMI), concurrent use of ≥2 hypertensive medications, presence of atrial fibrillation, pulmonary hypertension (pulmonary artery pressure >35 mmHg), >60 years of age, and an E/e′ > 9.^24^ The HFA-PEFF score uses four distinct steps in aiding the diagnosis of adult HFpEF. Step 1 is completing a pretest assessment that includes signs and symptoms, comorbidities, an ECG and echocardiogram, measurement of natriuretic peptides, and a 6-min walking test. Step 2 includes comprehensive echocardiography and natriuretic peptides if not measured previously. Step 3 includes an exercise stress test as well as invasive hemodynamic measurements. Step 4 includes CMRI, biopsies, scintigraphy, genetic testing, and specific laboratory testing.^25^ While both algorithms have been shown to be predictive of a HFpEF diagnosis, these algorithms have not been shown to be reliably extrapolated to pediatric populations. Despite this, imaging modalities such as electrocardiograms and echocardiograms, natriuretic peptide markers, genetic screenings, and prior medical history and other clinical assessment tools are all utilized to make a diagnosis of HFpEF in a pediatric patient. While a unified diagnostic algorithm is still absent regarding the diagnosis of pediatric HFpEF, evolving technology may bring advancement and new insights into this challenging diagnosis.

In adults, HFpEF generally remains a preserved EF phenotype, although a subset of patients experience declines in systolic function over time. Longitudinal registry and community cohort studies indicate that approximately 18% of HFpEF patients transition to HFrEF and ~21% to mid-range EF, with greater declines observed in older adults and those with coronary artery disease, and subclinical ventricular dysfunction may worsen even when EF is preserved.^26–28^ Overall, progressive systolic impairment is uncommon, and preserved EF remains the predominant trajectory. In pediatric HFpEF, longitudinal data are lacking, and it is unclear whether children exhibit similar potential patterns of change.

The range of clinical, imaging, and developing modalities used to assess HFpEF is compiled in Table 1. Clinical signs and symptoms remain crucial, although they are frequently subtle or non-specific in young individuals. Compared to traditional indices, echocardiography—specifically tissue Doppler and strain imaging—offers higher sensitivity; nonetheless, interpretation necessitates age-specific reference values. Although they lack established pediatric cutoffs, biomarkers like NT-proBNP and indicators of inflammation and fibrosis provide objective or exploratory insights. Although additional pediatric validation is required, novel techniques such as circulating non-coding RNAs and AI-based ECG/echocardiographic models offer promise for more accurate phenotyping and early identification.

### Emerging AI-based approaches

The development and incorporation of artificial intelligence (AI) in all aspects of life, including medicine, has risen exponentially in recent years. With no exception, novel artificial intelligence algorithms, specifically Convolutional Neural Networks (CNN), have been explored in the diagnosis of HFpEF in comparison to traditional diagnostic criteria. In 2020, Kwon et al. were the first to utilize an AI model for predicting HFpEF. They used a Deep Learning Model with ECG and clinical data to predict HFpEF in adult populations, finding that it demonstrated reasonable performance and disease detection.^29^ In 2021, Unterhuber et al. utilized the first CNN to identify patients with HFpEF using the European Society of Cardiology guidelines at the time and ECG data. They found that the CNN was reliable in identifying patients at risk for HFpEF, and externally validated the model by testing it against known HFpEF patients.^30^ More recently, in 2025, Akerman et al. used a CNN AI model to detect HFpEF in adults using echocardiographic videos. The model was tested against the H_2_FPEF scoring system used to diagnose HFpEF in symptomatic euvolemic patients. The study found that the model resulted in more informative data available for diagnosis, which led to fewer “intermediate” classifications of disease, more definitive diagnoses, and increased sensitivity and specificity.^31^ The combination of AI and clinical score information resulted in greater rates of appropriate treatment for HFpEF and fewer unnecessary treatments. This highlights the synergistic potential of combining AI and standard clinical practice to enhance positive patient outcomes.

AI applications have begun to extend into pediatrics, albeit still in the early stages of development. In 2021, Arnaout et al. used an AI model to screen fetal ultrasounds of the heart and predict CHD, a common etiology of pediatric HFpEF. They reported a sensitivity of 95% and a specificity of 96%, with a 100% negative predictive value. The model’s sensitivity was comparable to that of clinicians, supporting the conclusion that similar AI models could be used to enhance the detection of fetal CHD.^32^ Most recently, Nogimori et al. conducted the first study using an AI model to predict HF (regardless of EF) in a pediatric population. The study utilized 21,378 electrocardiograms from 8324 children to train a CNN using BNP levels and the occurrence of major adverse cardiovascular events (MACE) as outcome labels. The model generated an electrical HF indicator, which was then tested on 813 ECGs from 295 children. The model was found to predict MACEs with 1.6–7.5 times higher precision than BNP in the early period. This study showcased the potential for AI models to assist clinicians in diagnosing pediatric HF.^33^ Additionally, Mayourian et al. developed an AI-enhanced ECG model to detect left ventricular hypertrophy in pediatric patients without major CHD, using a large internal cohort and an external validation set. The AI model demonstrated superior diagnostic accuracy compared to that of expert interpretations by pediatric cardiologists. Adding clinical variables, such as age and sex, to the model yielded only marginal gains, suggesting that the AI model directly captures key structural cardiac features from ECG signals.^34^ These results highlight the potential of AI to improve early, non-invasive detection of ventricular remodeling in children. While clinical application in pediatric populations remains in its early stages, emerging evidence suggests promising potential for advancing the diagnosis of HFpEF using AI.^35^ To translate these tools into clinical practice, future efforts should focus on developing large, diverse, pediatric-specific datasets, as well as multicenter validation of existing models. Additionally, integrating AI outputs with clinical decision-making tools and electronic health records can support more individualized care pathways. Eventually, AI-based approaches may support diagnostic strategies as pediatric-specific data resources expand.

## LONG-TERM OUTCOMES FOR PEDIATRICS HFPEF

### Obesity-related HFpEF

It is widely recognized that obesity increases the risk of death from cardiovascular disease and all causes. Twig et al. specifically examined the correlation between BMI in adolescents and long-term outcomes, including cardiovascular death, in adulthood. Taking over 2.3 million patients and 42,297,007 person-years of follow-up, the group reported a significant increase in the risk of cardiovascular disease, stroke, coronary artery disease, and all-cause mortality for patients above the 50th percentile in BMI in adolescence. This risk exponentially rose with increasing BMI.^36^ Regarding obesity-related diastolic dysfunction in pediatrics, several studies have tracked the changes associated with weight reduction in obese patients. Ippisch et al. investigated the changes in cardiac parameters resulting from weight loss achieved through bariatric surgery. The post-surgery patients had a significant decrease in LVH as well as a significant increase in LV geometry and diastolic function parameters such as the E/A ratio.^37^ Inge et. al. et al. found similar results in a study comparing patients 3 years after bariatric surgery, with significant improvements reported in cardiometabolic health factors, such as hypertension and dyslipidemia, as well as improvements in quality of life.^38^ Lastly, a 9-year follow-up study on sleeve gastrectomy by Elhag and El Ansari demonstrated further improvements in cardiometabolic risk factors, including systolic and diastolic blood pressure, and LDL cholesterol.^39^ Regarding weight loss specifically through diet and exercise, Obert et al. reported a reversal of longitudinal strain through weight loss involving diet and exercise in adolescents.^40^ Lastly, Erbs et al. found that weight loss in obese pediatric patients returned global longitudinal strain to baseline values comparable to those of normal-weight patients, as well as a significant decrease in the left ventricular mass index compared to obese patients.^41^

Individual methods of weight loss, including specific diets and exercise, have shown mixed results in improving cardiometabolic health, particularly in pediatric populations. However, the overall benefit of returning to normal BMI during adolescence and its effect on health outcomes in adulthood has been reported. Juonala et al. analyzed four prospective studies on the long-term outcomes of weight loss in adolescence and reported that patients who were obese in childhood but nonobese as adults had the same cardiovascular risk as patients who maintained a normal BMI in both childhood and adulthood.^42^ Long-term data on the change in diastolic dysfunction and associated health outcomes regarding weight loss in pediatric patients are limited, but data suggest that the earlier weight loss occurs, the better the long-term outcomes related to cardiovascular health.^43^

Adult studies relating to weight loss and diastolic dysfunction have also been reported. One such study found that patients undergoing weight loss experienced a significant decrease in left ventricular mass, end-diastolic volume, and stroke volume, along with an increase in peak ventricular filling rate.^44^ The question may arise as to how the severity of obesity affects the prognosis of diastolic dysfunction/HFpEF, as well as the potential for reversal. In 1995, Alpert et al. published data examining the impact of longevity on cardiac physiology associated with obesity, as well as the effects of weight loss on adult patients. The group reported that as the duration of obesity increased, so did the association of hypertension, LV mass/height, LV internal dimension in diastole, and E-wave deceleration. Conversely, the duration of obesity was associated with a further decrease in LV fractional shortening and E/A ratio. Interestingly, there was also a significant association between the duration of obesity and the weight loss-induced change in all the described parameters.^45^ Nakajima et al. also found that as the duration of obesity increased in adults, the LV enlargement and wall thickening associated with cardiac dysfunction increased as well.^46^ Lastly, the CARDIA study examined the effects of obesity in normal-weight 18–30-year-old patients, measuring them at follow-up intervals of up to 25 years. The study found that in participants who became obese during the follow-up period, a longer duration of obesity from young adulthood into adulthood was associated with significant increases in LV mass and lower LV function in midlife.^47^

While pediatric data regarding long-term outcome studies are limited, the evidence in adults suggests that early, aggressive treatment is beneficial for combating obesity-related diastolic dysfunction. A longer duration of obesity, as well as an earlier age of onset, can lead to even more detrimental effects in a patient’s life. With the exponential increase in pediatric obesity being recorded in the United States, effective medication and lifestyle interventions should be further studied to prevent lifelong alterations to cardiovascular function and general health.

### Single ventricle

While obesity is a significant modifiable risk factor in the development of diastolic dysfunction and ultimately HFpEF, pediatric patients exhibit a heterogeneous collection of etiologies upon presentation. One such etiology is the single ventricle physiology. Patients presenting with a single ventricle can have left ventricular or right ventricular dominance, and while single ventricle palliation in general has impaired survival compared to the general population, patients with single right ventricles RV have been shown to have further impaired survival post-Fontan palliation.^48^ Specifically, one retrospective study found that a 20-year follow-up survival rate for LV-dominant patients was 72% compared to 52% for RV-dominant patients.^49^ Atz et al. reported a 90% survival rate at 11 years post operation, but noted a significant decrease in EF, exercise tolerance, and overall cardiovascular function compared to baseline.^50^ These findings underscore the need for lifelong monitoring and management of Fontan patients.

## PHARMACOLOGIC THERAPIES

The heterogeneous etiology of pediatric HFpEF has posed challenges to the development and investigation of targeted therapeutic approaches. As a result, consensus guidelines for treatment are also scarce. In 2013, the Canadian Cardiovascular Society published guidelines on the presentation, diagnosis, and medical management of HF in children. However, the authors noted that diastolic HF, while an important topic, is poorly understood and is accompanied by limited evidence for treatment. Therefore, it was excluded from the scope of the guidelines.^51^ The following year, the International Society for Heart and Lung Transplantation (ISHLT) published guidelines regarding the management of pediatric HF. These guidelines state that diuretics are recommended to establish an euvolemic state and potentially treat hypertension in children with HFpEF (level of evidence C). ACE inhibitors and other RAAS system blockades were only indicated if hypertension was present. In addition, the following medication classes were shown with a C level of evidence: calcium channel antagonists, phosphodiesterase inhibitors, and digoxin. Mineralocorticoid/aldosterone receptor antagonists (spironolactone or eplerenone), positive inotropic agents (dopamine, dobutamine, etc.), and the usage of pulmonary vasodilators (prostaglandins) were not recommended for use in pediatric HFpEF with a class level of evidence C. While these guidelines provided clinicians with insight into treatment options and contraindications for pediatric HFpEF patients, the available options and supporting evidence remained limited.^51^ In 2024, the American Heart Association published a statement regarding the evaluation and management of chronic HF in children with CHD. However, treatment options specifically for CHD patients with preserved EF were absent.^52^ Most recently, a 2025 update to the 2014 ISHLT guidelines was released. The consensus treatment for pediatric HFpEF in biventricular patients is diuretics and sodium-glucose cotransporter-2 inhibitors (SGLT2i) (level of evidence C). It was stated that the guideline-directed medical therapy (GDMT) for adult HFpEF treatment could be applied to children with biventricular circulation and a systemic LV regardless of HF etiology.^53^

The most recent iteration of GDMT for adult HF is from the AHA in 2022. First-line therapy is recommended to be diuretics as needed, followed by SGLT2i (class 2a), angiotensin receptor-neprilysin inhibitors (ARNi) (class 2b), mineralocorticoid receptor antagonists (MRA) (class 2b), and angiotensin receptor blockers (ARB) (class 2b). All 2b recommendations were directed to be in greater benefit to patients with left ventricular ejection fraction (LVEF) closest to 50%.^54^ In the absence of rigorous studies demonstrating efficacy in pediatric populations, physicians are left to rely on experience and extrapolate adult therapy for pediatric management.

### Diuretics

The first-line therapy for HFpEF treatment in all age groups is indicated for controlling preload and afterload on the heart. The AHA adult HF guidelines state that furosemide or other loop diuretics, which inhibit the reabsorption of sodium or chloride, are the preferred first-line diuretic for fluid management in HF. Thiazide diuretics, such as hydrochlorothiazide or chlorthalidone, may be added to the treatment regimen if additional fluid removal is needed or if patients are not responding to a moderate to high dose of loop diuretics. Diuretic usage in adult HF patients is associated with improvement in symptoms, quality of life, and exercise tolerance, in addition to a lowering 30-day all-cause mortality after discharge from the hospital vs no diuretic use.^54,55^ Minimal published data support the use of diuretics in pediatric HF; however, it has been shown that low loop diuretic responsiveness in pediatric HF patients is correlated with worse outcomes and extended hospital stays.^56^

### Mineralocorticoid receptor antagonists

Potassium-sparing diuretics, or MRAs, remain a contested treatment modality for pediatric and some adult HF. A 2024 meta-analysis of four trials, RALES (Randomized Aldactone Evaluation Study), EMPHASIS-HF (Eplerenone in Mild Patients Hospitalization and Survival Study in Heart Failure), TOPCAT (Treatment of Preserved Cardiac Function Heart Failure with an Aldosterone Antagonist), and FINEARTS-HF (FINerenone trial to investigate Efficacy and sAfety superioR to placebo in paTientS with Heart Failure) looked at the efficacy of MRAs in HF. The effectiveness of MRAs in adult HFrEF in reducing HF hospitalization, cardiovascular death, and all-cause death was well elucidated. However, for HFpEF, MRAs were only shown to be significant in reducing the number of heart failure hospitalizations.^57^ It is noteworthy that the TOPCAT trial found a decreased rate of both hospitalization and cardiovascular death in HFpEF patients treated with MRAs from the Americas, specifically. However, the authors cautioned that post hoc analyzes of any clinical trial should be viewed with caution.^58^ Similarly, the Aldo-DHF trial demonstrated that spironolactone improved diastolic function, exercise capacity, heart failure symptoms, and quality of life in adult patients with HFpEF.^59^

Aldosterone contributes directly to adverse cardiac remodeling, including myocardial fibrosis, hypertrophy, and dysfunction, independent of its effects on blood pressure.^60^ In patients with HFpEF, an increase in serum aldosterone levels has prognostic significance and is correlated with changes in ventricular geometry.^61^ In addition, a combination of diabetes and elevated aldosterone (mineralocorticoid excess) acts synergistically to promote HFpEF in experimental animal models.^62,63^ In 2024, Solomon et. al. reported a double-blind trial using finerenone in the treatment of adult HFpEF, a nonsteroidal, selective MRA. Finerenone resulted in a significantly lower rate of worsening heart failure events and death from cardiovascular causes.^64^ In pediatric patients presenting after CHD procedures with HFpEF, a 2013 study by Masutani et al. found that aldosterone was significantly elevated compared to elevations in BNP seen in systolic heart failure patients.^65^ While no studies to date have been published regarding the efficacy of MRA treatment in pediatric HFpEF, future studies could shed light on the pathophysiology and potential benefits of the medication in this population. It should be noted that the routine use of MRAs in pediatric HFpEF is not recommended per the 2025 ISHLT guidelines.

### Renin angiotensin system modulation

The other adult GDMT for the treatment of HFpEF are ARBs and ARNi. The CHARM (Candesartan in Heart failure: Assessment of Reduction in Mortality and morbidity)-Preserved trial found that candesartan treatment in adult HFpEF patients prevented hospitalization at a significant rate.^66^ However, the I-PRESERVE (Irbesartan in Heart Failure with Preserved Ejection Fraction) study found that adults treated with irbesartan did not have improved outcomes, and it failed to reduce the hospitalization rate from a cardiovascular cause.^67^ Regarding ARNi use, the PARAGON-HF found that sacubitril-valsartan did not result in a significantly lower rate of total hospitalizations for adult HFpEF patients, in addition to no benefit in patients prescribed valsartan, an ARB.^68^ While the PANAROMA-HF study that led to FDA approval for ARNi use in pediatric HFrEF demonstrated efficacy of the medication, these studies have not been replicated in HFpEF patients.^69^ In patients with CHD, pediatric patients in one single-center study showed some improvements in ventricular function, while also recording high rates of hypotension and adverse events.^70^ While pediatric HFrEF has more conclusive guidelines for therapy,^53^ more studies are needed to elucidate the potential use of ARB or ARNi therapy in pediatric HFpEF patients.

### SGLT2_i_

SGLT2 inhibitors are primarily used to treat type 2 diabetes mellitus (T2DM) by inhibiting glucose reabsorption in the nephrons of the kidney. However, in recent years, studies have looked at the efficacy of this drug class for the treatment of HF. Trials such as Dapagliflozin Evaluation to Improve the Lives of Patients with Preserved Ejection Fraction Heart Failure and Empagliflozin in Heart Failure with a Preserved Ejection Fraction looked into the efficacy of SLGT2i treatment on HFpEF specifically. The former found that dapagliflozin reduced the combined risk of worsening HF or cardiovascular (CV) death among patients with mildly reduced or preserved EF.^71^ Similarly, the EMPEROR-Preserved trial demonstrated that empagliflozin reduced the combined risk of CV death or hospitalization for HFpEF patients with or without diabetes.^72^ Additionally, the EMPEROR-Preserved (Empagliflozin Outcome Trial in Patients with Chronic Heart Failure with Preserved Ejection Fraction) trial found that empagliflozin was equally beneficial in HFpEF, regardless of blood pressure modifications. The results of clinical trials examining the efficacy of SGLT2i medication in adult HFpEF show promising outcomes. In recent years, smaller studies have been conducted to investigate the potential effectiveness of SGLT2i in pediatric HFpEF populations.

The use of SGLT2i in pediatric HF cases has become more common, though research is still limited on the efficacy of the treatment in pediatric populations. Safety studies related to the use of dapagliflozin and empagliflozin in pediatric diabetes and other diseases indicate that these medications are well-tolerated in children.^73–76^ Newland et al. published data suggesting that dapagliflozin use in pediatric cases of advanced HF awaiting transplant was not associated with perioperative adverse events, such as death, or associated with increased length of hospitalization post-transplant.^77^ In terms of efficacy, several smaller studies with regard to dapagliflozin specifically have been reported recently. In 2022, Newland et al. conducted the first pediatric study of dapagliflozin in a pediatric population with HF.^78^ Thirty-eight pediatric HF patients underwent the addition of dapagliflozin to their standard HF therapy. While this study primarily targeted pediatric HFrEF, the medication was deemed to have a reasonable safety profile, along with evidence of increasing LVEF in patients taking the drug. Kumar et al. conducted a small observational study that incorporated dapagliflozin into pediatric HF treatment, demonstrating an increase in LVEF, improvement in symptom profile, NYHA class, and NT-proBNP levels.^79^ Most recently, in 2025, Butts et al. published on the usage of SGLT2i in pediatric HF (most with decreased ventricular systolic function). The medication was found to be well-tolerated, with improvements in both EF and BNP in the pediatric patients.^80^

While data and trials on the pediatric use of SGLT2i for HF, and particularly HFpEF, are very limited, evidence from pediatric patients with CHD suggests a good safety profile with an efficacious result, as noted by a decrease in NT-pro BNP or BNP.^81^ However, as noted by Butts et al., the lack of commercially available liquid formulations poses a limitation in pediatric usage due to the inability to swallow pills. In addition, insurance coverage for SGLT2i prescriptions in pediatric HF patients is limited.^80^ Despite the limited pediatric data and lack of clinical structure in the administration of the medication class, SGLT2i are being increasingly incorporated into the practice of pediatric HF centers as a 4th line agent.^82^ While studies are still lacking in relation to pediatric HFpEF intervention with SGLT2i, the drug class received FDA approval for treating pediatric T2DM. Empagliflozin gained approval following the DINAMO (Diabetes Study of Linagliptin and Empagliflozin in Children and Adolescents) trial (NCT03429543), where the medication provided clinically significant reductions in A1c (a placebo-corrected A1c reduction of at least 0.5% was considered clinically relevant).^74^ Similar results were reported for dapagliflozin, which was FDA-approved following the T2NOW trial (NCT03199053).^75^

### GLP-1 receptor agonists (RA)

In 2024, a meta-analysis examining HF-related outcomes in adult patients with and without HF found that GLP-1 RA did not reduce HF hospitalization for those with a history of HF. However, it lowered this risk in patients without a history of HF. Furthermore, GLP-1 RA consistently reduced ischemic events regardless of the presence of HF.^83^ The STEP-HFpEF trial was included in the analysis that specifically targeted HFpEF. It showed that GLP-1 RA usage in HFpEF patients resulted in larger reductions in symptoms and physical limitations, along with greater improvements in exercise and weight loss compared to placebo.^84^ Additionally, the semaglutide group experienced a significant decrease in CRP, a marker of inflammation linked to increased comorbidity burden in HFpEF.^85^ The SELECT (Semaglutide Effects on Cardiovascular Outcomes in People with Overweight or Obesity) Trial further demonstrated that GLP-1 RA therapy in patients with cardiovascular disease and obesity without diabetes reduced cardiovascular mortality.^86^ In 2024, the SUMMIT (Study of Tirzepatide in Participants With Heart Failure With Preserved Ejection Fraction and Obesity) trial reported that the use of tirzepatide decreased CV death and worsening HF, and improved KCCQ-CSS score compared to placebo.^87^ A subsequent cardiac magnetic resonance (CMR) study on 106 participants with obesity-related HFpEF from the parent SUMMIT trial revealed a decrease in LV mass and a decrease in paracardiac adipose tissue compared to the placebo cohort. It was proposed that these physiologic changes could contribute to the reduction in HF events that the parent SUMMIT trial reported.^88^ A study in 2024 published by Patel et al. looked at the additive benefit of GLP-1 RA combined with SGLT2i in obese patients with T2DM and HFpEF. They concluded that the addition of GLP-1 RA to a SGLT2i regimen significantly reduced the risk of HF exacerbations, all-cause emergency department hospitalizations, new onset arrhythmia, new onset acute kidney injury, and pulmonary hypertension when compared to treatment with SGLT2i alone.^89^

The use of GLP-1 RA in pediatrics is a relatively recent and emerging area. Liraglutide and semaglutide were FDA-approved for the treatment of obesity in adolescents aged 12–17 years.^90^ Individual trials, such as that conducted by Kelly et al., have found that adolescents taking liraglutide in addition to lifestyle therapy experienced a greater reduction in BMI compared to those taking a placebo plus lifestyle therapy.^91^ Fox et al. reported similar conclusions in a randomized controlled trial that included pediatric patients from a younger age, 6–12 years, showcasing the preliminary efficacy of GLP-1 RA in populations younger than those currently approved by the FDA.^92^ Beyond individual trials, meta-analyzes have consolidated the emerging evidence base on the use of GLP-1 RA in pediatric populations. In 2021, a meta-analysis of nine randomized controlled trials involving 574 children and adolescents with obesity or type 2 diabetes demonstrated that GLP-1 RAs were generally safe and effective in modestly reducing weight, BMI, and systolic blood pressure, with nausea as the most common adverse effect. Glycemic control improved significantly, especially in children with evidence of insulin resistance.^93^ Kotecha et al. further expanded this evidence by analyzing 18 RCTs involving over 1400 children aged 6–17 with obesity, prediabetes, or type 2 diabetes. Their findings reaffirmed significant benefits in weight, BMI, HbA1c, and systolic blood pressure, with no new safety concerns.^94^

While GLP-1 receptor agonists have shown promising effects in adult HFpEF populations, particularly those with obesity and metabolic syndrome, pediatric data are primarily confined to weight loss trials in obese children. Their cardiovascular effects in pediatric HF remain unexplored. Given their metabolic benefits, GLP-1 RAs may offer a promising therapeutic avenue, especially in obesity-related HFpEF; however, robust pediatric-specific cardiovascular studies are still lacking.

## INVESTIGATIONAL THERAPIES

### Cellular therapies

Stem cell therapy has become increasingly utilized over the past decade, with advancements in treatment options and an expansion of knowledge in the field. The following approaches remain investigational and are not part of routine clinical management.

Hypoplastic left heart syndrome (HLHS) is a rare congenital heart defect characterized by the underdevelopment of the left heart. The absence of a fully functional left ventricle in HLHS forces the right ventricle (RV) to take on the majority of the circulatory workload. This chronic hemodynamic burden induces RV hypertrophy, fibrosis, and diastolic dysfunction, resembling HFpEF.^95^ The TICAP (Transcoronary Infusion of Cardiac Progenitor Cells in Patients with Single-Ventricle Physiology) phase I trial and PERSEUS Phase II trials delivered intracoronary cardiosphere-derived cells (CDCs) in HLHS, demonstrating safety and improvements in cardiac function.^96,97^ The APOLLON (Cardiac Stem/Progenitor Cell Infusion in Univentricular Physiology) Phase III trial (NCT02781922) is currently underway. ELPIS (Allogeneic Human Mesenchymal Stem Cell Injection in Patients with Hypoplastic Left Heart Syndrome) Phase I trial using mesenchymal stem cells (MSCs) demonstrated safety and potential RV benefit, with follow-up trials ongoing (NCT03525418) (NCT04925024) (NCT03406884). The POSEIDON (Percutaneous Stem Cell Injection Delivery Effects on Neomyogenesis) trial in nonischemic dilated cardiomyopathy showed that allogeneic MSCs were safe and effective.^98^

Wittenberg et al. studied allogenic mesenchymal precursor cells (MPCs) showing improvement in left ventricular volume over 12-months.^99^ Burkhart et al. found that autologous umbilical cord blood-derived mononuclear cells (UCB-MNC) injections were safe but did not enhance cardiac function in HLHS patients undergoing stage II palliation.^100^ BM-MNC therapy after Fontan appears safe with mild right ventricular EF improvement.^101^ CD34^+^ cells therapy in adults with cardiomyopathy or HFpEF improved diastolic function, NT-proBNP, and exercise capacity.^102,103^

Stem cell therapies represent a frontier approach for pediatric heart failure, particularly in structural conditions like HLHS. While safety and feasibility have been established across several early-phase trials, results on functional improvement are mixed, and benefits are often modest or delayed. Many studies have small cohorts and heterogeneous endpoints, which limit the ability to draw broad conclusions. Nevertheless, continued interest in regenerative strategies suggests the need for larger, controlled trials that standardize cell type, delivery method, and patient selection.

### Neuromodulation therapy

Neuromodulation therapy is an alternative to HFpEF treatment that targets the autonomic imbalance of the body that may occur during HFpEF. There are two primary routes of therapy targeting the autonomic nervous system that have been studied in HFpEF: Baroreflex Activation Therapy (BAT) and Non-Invasive Vagus Nerve Stimulation (nVNS). While both modes of therapy aim to improve cardiac function and markers in heart failure, they do so via different though complementary mechanisms.

BAT inhibits the sympathetic nervous system while enhancing the parasympathetic nervous system, thereby mitigating the harmful autonomic effects seen in HF. This is accomplished by stimulating the carotid sinuses with an implanted device. Linde et al. reported that the use of BAT in HFrEF led to significant improvement in quality of life, 6-min walking time, and NYHA functional class.^104^ A study conducted by Schafer et al. studied BAT in patients with treatment-resistant hypertension (with and without HFpEF), finding that BAT therapy significantly reduced systolic and diastolic blood pressure, decreased posterior wall diameter and LV mass, and increased e’ lateral velocity significantly.^105^

Sabbah et al. utilized a canine model of coronary microembolization-induced heart failure to study the chronic effects of BAT over 3 months. They reported a significant decrease in LV hypertrophy and interstitial fibrosis. The improvement in structure was associated with reduced sympathetic nerve activity (SNA) and a normalization of β1-adrenergic receptors and nitric oxide synthase, suggesting a mechanistic link to autonomic modulation.^106^ A study by Clemmer and Pruett reported the use of HumMod, a human physiology computer model, to investigate the effects of BAT on HFpEF physiology over a 6-month simulation period.^107^ The study found that BAT significantly reduced systolic blood pressure while mostly preserving cardiac output and increasing EF, as well as increasing plasma volumes despite reducing cardiac volumes and pressures. When cardiac SNA was not allowed to decrease during the BAT simulations, LV hypertrophy improvements were impaired compared to SNA suppression. Clemmer and Pruett thus concluded that the reductions in cardiac SNA during BAT are crucial and primarily responsible for the improvements in cardiac function and hypertrophy.^107^ However, there is no clinical validation yet for this computational study.

Within the invasive neuromodulation therapy space, the ANTHEM-HFpEF Study was the first to investigate the safety and feasibility of autonomic regulation therapy in patients with HFpEF or HFrEF. An electrical lead system was placed around the right cervical vagus nerve of patients, and results were measured at a 12-month follow-up. At the follow-up, NYHA class, 6-min walk distance, and quality of life were improved. Patients also saw a 29% decrease in low-frequency/high-frequency heart rate variability. The study concluded that the intervention was safe and well-tolerated in HFpEF or heart failure with mildly reduced ejection fraction (HFmrEF) patients and was associated with an improvement of symptoms, stability, and autonomic tone.^108^

nVNS stimulates the auricular branch of the vagus nerve by sending electrical pulses in a transcutaneous fashion on the surface of the ear. Clancy et al. found that nVNS can increase heart rate variability (HRV) and reduce sympathetic nerve outflow in healthy subjects, an effect desirable in HF physiology.^109^ In 2017, Antonino et al. found that resting heart rate and HRV decreased, while cardiac baroreflex sensitivity increased, during nVNS therapy in healthy men, furthering the potential outlook for nVNS in cardiovascular modulation.^110^ In 2025, Ackland et al. demonstrated that nVNS improves measures of cardiorespiratory fitness and reduces inflammation in a cost-effective and safe approach to enhancing exercise capacity in healthy individuals.^111^

Within animal models, several HFpEF studies have been undertaken to investigate the potential efficacy of nVNS therapy in the disease physiology. Zhou et al. found that in rat HFpEF models, intermittent nVNS therapy could significantly improve diastolic dysfunction and lower inflammation and fibrosis.^112^ In 2022, Elkholey et al. described how NVNS usage in rat HFpEF models improved cardiac function through anti-inflammatory and anti-fibrotic effects.^113^

In HFpEF populations, Tran et al. reported that nVNS therapy in twenty-four participants improved longitudinal strain and HRV in patients with diastolic dysfunction.^114^ Stavrakis et al. corroborated the report of improvement in longitudinal strain, as well as found that nVNS therapy reduced inflammatory cytokine levels and increased the quality of life in HFpEF patients.^115^

Research on neuromodulation in pediatric populations has primarily focused on conditions like epilepsy and migraine. nVNS is FDA-approved as an adjunctive therapy for pediatric patients aged 12 years and older. The approval was based on studies demonstrating its ability to reduce seizure frequency and improve quality of life in pediatric patients with epilepsy who were not candidates for surgery. Studies have shown that nVNS can increase response rate, reduce seizure frequency, and improve quality of life in children, and highlighted its safety profile.^116^ Similar benefits have been observed in pediatric migraine patients, particularly with low-frequency non-invasive auricular vagus nerve stimulation.^117^ However, neuromodulation in pediatric HF remains largely unexplored.

### Pericardiotomy

The membranous sac surrounding the heart, known as the pericardium, exerts a compressive force on the surface of the heart. This force is amplified when the heart volume increases during diastole. While the pericardium normally acts as a barrier to the heart and an anchor to maintain its position, it can also play roles in the pathophysiology of HFpEF. The compressive force exerted by the pericardium on the heart can become exaggerated in various forms of heart failure.^118^ As found by Obokata et al., obesity-related HFpEF patients exhibit greater pericardial restraint due to an increase in epicardial heart volume.^119^ With obesity being a common cause of pediatric HFpEF,^12^ as well as constrictive pericarditis, treatment involving the pressure placed on the heart in HFpEF has intriguing possibilities. A study done by Borlaug et al. in 2017 provided a proof of concept by performing a pericardial resection on a pig model of HFpEF. The results showed a mitigation in the elevation of LV filling pressures with volume loading, a simulation of the hemodynamic loading seen with exercise.^120^ Following this, Borlaug et al. performed the first human study on the effects of surgical pericardiotomy on treating HFpEF. An anterior pericardiotomy was performed on four female patients, who all presented with the obesity phenotype of HFpEF. The study showed a significant mitigation of the rise of LV filling pressures during volume loading after the operation and a 67% decrease in the pulmonary capillary wedge pressure (PCWP).^121^ Additionally, patients without a diagnosis of HFpEF showed no effect of the pericardiotomy on baseline PCWP. There were no major cardiovascular, renal, or cerebrovascular events at 6 months of follow-up. Further testing to assess the efficacy and safety of minimally invasive pericardiotomy for the treatment of HFpEF is ongoing, including the clinical trial, the PeriCut Catheter System Early Feasibility Study (REIMAGINE-HFpEF, NCT06702501).^122^

While still in the early stages of study, the initial evidence from surgical anterior pericardiotomy shows promise in the management of HFpEF. As such, there have been no studies of this treatment in children, and the PeriCut clinical trial only includes patients over the age of thirty. Despite this, the overlap of treatment indications, obesity, and constrictive pericarditis-related HFpEF lends acknowledgment to a potential future with treatment for pediatric HFpEF.

**Fig. 1:**
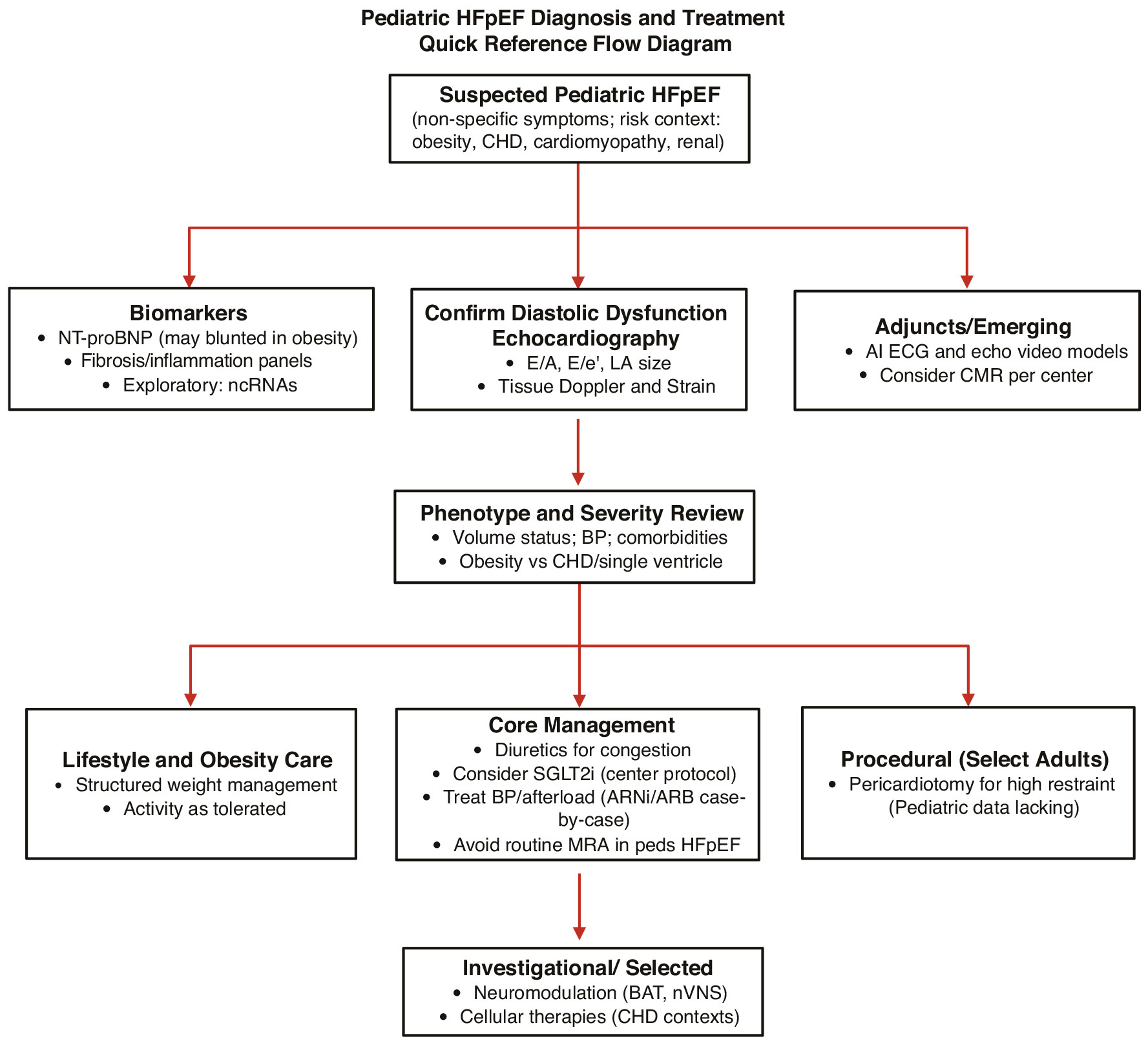
Diagnostic and therapeutic workflow for pediatric HFpEF. Assessment of phenotype and severity informs management strategies, including the use of diuretics for congestion, selective application of SGLT2 inhibitors or ARNi/ARB, and the avoidance of routine mineralocorticoid receptor antagonists. Lifestyle and weight management are crucial for obesity-related HFpEF. In contrast, investigational strategies such as neuromodulation and cellular therapies, along with procedural options like pericardiotomy, are still in the experimental phase. The figure is not intended to serve as a precise clinical guide, as tissue Doppler imaging values vary with age and cardiac growth in children, as do NT-proBNP/BNP values. Rather, the figure is intended to convey the rationale behind diagnosing and treating pediatric HFpEF.

The existing and new therapies for pediatric HFpEF are described in Table 2. Although there is insufficient trial data, SGLT2 inhibitors and GLP-1 receptor agonists, which are well-established in the treatment of adult HFpEF and obesity, are increasingly regarded as off-label in pediatrics. Loop diuretics remain the first-line treatment for congestion. Antihypertensive drugs like ARNi/ARB may be selectively beneficial in juvenile HFpEF; however, MRAs are not recommended because of inadequate efficacy data and safety issues. Weight and lifestyle management is the mainstay of treatment for obesity-related HFpEF; this approach has long-term benefits but also has adherence problems. There is an urgent need for pediatric-specific clinical studies, as investigational approaches such as neuromodulation, pericardiotomy, and cellular therapies are still in the experimental stage and have limited or no evidence in pediatric populations. Linking diagnosis and therapeutic options is critical to better treatment outcomes (Fig. 1).

### Conclusions and future directions

Heart failure with preserved ejection fraction and diastolic dysfunction are still underdiagnosed, but are becoming more significant causes of cardiovascular morbidity in children. Although the field of heart failure with preserved ejection fraction has seen rapid advancements, including improved diagnostic algorithms, driven by biomarker identification, risk-based stratification, and new pharmaceutical options, there has been limited application of these discoveries to children. Congenital cardiac disease, restrictive cardiomyopathy, cancer treatment, HIV, obesity, and renal disease are some of the causes of pediatric HFpEF. Due to the lack of established pediatric-specific criteria, reference ranges, and therapeutic trials, this variation complicates diagnosis and treatment.

Advanced methods, such as strain imaging, tissue Doppler, and speckle tracking, hold promise for more sensitive detection, as conventional echocardiographic tests frequently fail to provide accurate evaluations. Circulating biomarkers may be helpful in adults, but they must be validated for pediatric populations, particularly in relation to obesity and related issues. Furthermore, early safety and feasibility studies are currently being conducted in children for pharmacotherapy such as SGLT2 inhibitors and GLP-1 receptor agonists, which have demonstrated incremental advantages in adults.

Additionally, cutting-edge approaches such as surgical pericardiectomy, neuromodulation, and stem cell therapy are still in the experimental stage, demonstrating the variety of possible treatment options. Last, but not least, long-term outcomes for patients with single-ventricle hearts and obesity-related heart failure with HFpEF highlight the long-term consequences of early diastolic dysfunction and the urgent need for prompt management.

Several clearly defined research priorities can further advance the field of pediatric cardiovascular health. First and foremost, it is critical to develop established, standardized diagnostic procedures for pediatric diastolic dysfunction and HFpEF that are tailored to specific age groups and populations. Creating normative ranges for biomarkers and imaging methods is a key aspect of this. Additional mechanistic studies should be conducted to further elucidate the cellular and molecular mechanisms underlying pediatric HFpEF, with a focus on obesity-related and congenital variations.

Multicenter pediatric clinical trials are urgently needed to assess the safety and efficacy of treatments currently adapted from the management of adult HFpEF patients. These therapies include SGLT2 inhibitors, GLP-1 receptor agonists, angiotensin receptor neprilysin inhibitors (ARNi), and mineralocorticoid receptor antagonists (MRAs). Moreover, the integration of artificial intelligence, advanced imaging techniques, and digital biomarkers into pediatric practice requires thorough evaluation, especially regarding multicenter validation and their incorporation into clinical decision-making frameworks.

Despite significant advancements in understanding the pathophysiology and management of adult HFpEF, a well-defined clinical framework for pediatric HFpEF remains lacking, highlighting the critical need for targeted research in this population. In summary, research on pediatric HFpEF requires a concentrated effort among clinicians, basic research scientists, and AI engineers. Progress in this area will require collaboration with various medical groups, standardization of diagnostic criteria, and a significant investment in developing pediatric-specific treatments. Bridging the gap between developments in adult medicine and the specific needs of pediatric patients, we strive toward evidence-based, customized care that enhances survival rates and quality of life for children with diastolic dysfunction and HFpEF.

## Figures and Tables

**Table 1.** Diagnostic tools and biomarkers in pediatric HFpEF.

Domain	Tool / Marker	Use in pediatrics	Strengths	Limitations
Clinical	Symptoms + exam (exertional dyspnea, tachycardia, hepatomegaly)	Often nonspecific; integrate with risk context (obesity, CHD, cardiomyopathies).	Universal, low-cost, repeatable.	Low specificity; may be absent at rest.
Echocardiography	Conventional Doppler (E/A, E/e′, LA size)	Interpret with age-specific ranges; LA enlargement suggests chronically elevated filling pressures.	Widely available; noninvasive.	Conventional indices have limited sensitivity in children; age dependency.
Echocardiography	Tissue Doppler / Speckle-tracking (e′, strain, strain rate)	Improves detection of impaired relaxation and stiffness beyond conventional indices.	More sensitive to early diastolic dysfunction.	Technique-dependent; pediatric reference ranges are still maturing.
Biomarkers	NT-proBNP / BNP	Useful for HF; levels may be lower in individuals with obesity; interpret in a pediatric context.	An objective marker of myocardial stress.	Blunted in obesity; pediatric cutoffs not standardized.
Biomarkers	Fibrosis/inflammation markers (e.g., galectin-3, CRP, IL-6, GDF-15)	Investigational for HFpEF phenotyping and risk stratification.	May track systemic pathways of HFpEF.	Limited pediatric validation; assay availability.
Emerging	Circulating non-coding RNAs	Early-stage research for fibrosis and HF severity.	Potentially specific molecular signatures.	Not yet clinically relevant; pediatric data are sparse.
Emerging	Al-augmented ECG / Echo video models	Early studies show promise for HF prediction and LV remodeling detection.	Scalable and noninvasive, it may outperform single biomarkers.	Require pediatric-specific datasets and external validation.

**Table 2.** Treatment approaches for children with HFpEF.

Class / Strategy	Pediatric position	Advantages	Cautions / Gaps
Loop diuretics (± thiazide)	First-line for congestion; 2025 pediatric HF guidance supports use in biventricular HFpEF	Symptom relief, euvolemia, improved exercise tolerance.	Limited pediatric HFpEF trial data; monitor renal function/electrolytes.
SGLT2i	Emerging in pediatric HF; supportive safety data from T2DM; increasing off-label use in centers	Adult HFpEF benefit ( ↓ HF events); weight/glycemic benefits; once daily.	Pediatric HFpEF efficacy not yet proven; pill-only formulations; variable coverage.
GLP-1 RA	Approved for adolescent obesity; HFpEF data adult-only (benefit in obesity-related HFpEF).	Weight loss; improved symptoms/exercise capacity in adult HFpEF; anti-inflammatory signal.	No pediatric HFpEF trials; GI side effects; injection/weekly dosing.
ARNi / ARB	Consider when hypertension/afterload issues, pediatric HFrEF evidence, and HFpEF are mixed in adults.	Potential ↓hospitalizations (some adult trials); blood pressure control.	Adult HFpEF trials mixed/neutral; pediatric HFpEF data lacking; monitor renal/K + .
Mineralocorticoid receptor antagonists (MRA)	Routine use not recommended for pediatric HFpEF in 2025 guidance.	Adult data: ↓HF hospitalizations (HFpEF) and mortality (HFrEF).	Hyperkalemia risk; limited pediatric HFpEF evidence; guideline caution.
Lifestyle/weight management	Core for obesity-related HFpEF; earlier intervention improves long-term outcomes.	Improves strain, LV mass, and cardiometabolic risk; durable benefits.	Adherence: variable response to diet/exercise alone; access to programs.
Neuromodulation (BAT, nVNS)	Investigational; pediatric HF data are minimal.	Signals for improved symptoms, strain, and autonomic tone (adult/animal).	Device/infrastructure; long-term safety/efficacy in children is unknown.
Pericardiotomy (anterior/percutaneous)	Early adult feasibility data only.	Can lower filling pressures during volume load in adult HFpEF.	No pediatric data; operative/device risks; narrow phenotype applicability.
Cellular therapies (CDC, MSC, progenitors)	Trials primarily in single-ventricle CHD; mixed functional outcomes.	Potential reverse remodeling; early safety established in select trials.	Heterogeneous endpoints; modest benefits; not HFpEF-specific.

## IMPACT:

- This article addresses pediatric heart failure with preserved ejection fraction (HFpEF), a condition that is emerging in the pediatric population. While HFpEF is a well-established and prevalent condition in adults, its prevalence in children remains underrecognized and poorly understood.
- This article highlights key challenges in diagnosing and managing pediatric HFpEF, including the absence of standardized diagnostic criteria, limited sensitivity of traditional diastolic function measures, and a lack of age-specific imaging biomarkers.
- In this up-to-date review, we discuss those limitations, emphasizing the need for greater research into its pathophysiology and therapeutic strategies.
